# Induction of Excessive Endoplasmic Reticulum Stress in the *Drosophila* Male Accessory Gland Results in Infertility

**DOI:** 10.1371/journal.pone.0119386

**Published:** 2015-03-05

**Authors:** Clement Y. Chow, Frank W. Avila, Andrew G. Clark, Mariana F. Wolfner

**Affiliations:** Department of Molecular Biology and Genetics, Cornell University, Ithaca, New York, United States of America; Ecole Normale Supérieure, FRANCE

## Abstract

Endoplasmic reticulum (ER) stress occurs when misfolded proteins accumulate in the lumen of the ER. A cell responds to ER stress with the unfolded protein response (UPR), a complex program of transcriptional and translational changes aimed at clearing misfolded proteins. Secretory tissues and cells are particularly well adapted to respond to ER stress because their function requires high protein production and secretory load. The insect male accessory gland (AG) is a secretory tissue involved in male fertility. The AG secretes many seminal fluid proteins (SFPs) essential for male reproduction. Among adult *Drosophila* tissues, we find that genes upregulated by ER stress are most highly expressed in the AG, suggesting that the AG is already undergoing high levels of ER stress due to its normal secretory functions. We hypothesized that induction of excessive ER stress in the AG above basal levels, would perturb normal function and provide a genetic tool for studying AG and SFP biology. To test this, we genetically induced excessive ER stress in the AG by conditional 1) expression of a misfolded protein or 2) knockdown of the UPR regulatory protein, BiP. Both genetic manipulations induced excessive ER stress in the AG, as indicated by the increase in *Xbp1* splicing, a marker of ER stress. Both models resulted in a large decrease in or loss of SFP production and male infertility. Sperm production, motility, and transfer appeared unaffected. The induction of strong ER stress in the insect male AG may provide a simple way for studying or manipulating male fertility, as it eliminates AG function while preserving sperm production.

## Introduction

The endoplasmic reticulum is a large organelle that is involved in many essential cellular functions. ER stress occurs when misfolded proteins accumulate in the ER. The cell responds to ER stress with the complex, evolutionarily conserved, signaling network known as the unfolded protein response (UPR) [1,2,3]. The UPR consists of three main signaling branches, IRE1, ATF6, and PERK. The ER chaperone BiP holds all three of these pathways inactive when misfolded proteins are absent. The UPR maintains the careful balance between the folding capacity of the ER and the accumulation of misfolded proteins. Depending on context, ER stress and induction of the UPR can improve or exacerbate disease processes [4]. In addition to its role in disease, the UPR has wide ranging roles in handling ER stress associated with normal function and development [5].

The ER is the major site for the translation and processing of secreted and membrane bound proteins. Because of their high protein production and secretion, secretory cells and tissues must handle higher than normal levels of misfolded proteins. This places a constant demand on their ER that has resulted in a specially adapted UPR in different types of secretory tissue, allowing these tissues to process high levels of proteins through the ER [5]. For example, to prevent the accumulation of misfolded proteins associated with high protein production, mammalian plasma cells induce the UPR prior to increases in secretion of immunoglobulin [6,7].

*Drosophila* is becoming a powerful tool for studying the role of ER stress and the UPR in health and disease. The recent development of ER stress-related genetic tools, including transgenic fluorescent markers of ER stress, provides new opportunities to study this pathway [8,9,10]. Indeed, studies in *Drosophila* have helped to elucidate mechanisms of ER stress-induced retinal degeneration [8,11,12,13] and other diseases involving ER stress [14,15,16,17]. Furthermore, tools for studying natural genetic variation in *Drosophila* enabled the first study to identify the effects of genetic variation on the *in vivo* ER stress transcriptional response [18].

The *Drosophila* male accessory gland (AG) is a major reproductive tissue. The AG produces seminal fluid proteins (SFPs) that are secreted into the gland’s lumen and then transferred to the female during mating [19,20]. The presence and levels of SFPs are critical to normal reproductive success because SFPs influence the female in many ways, including altering her ovulation rate, feeding behavior, remating rate, egg production, and immunity [19,20]. The AG consists of two types of secretory cells: main cells and secondary cells [21], which secrete a suite of overlapping and unique SFPs [21,22]. AG cells are constantly producing and secreting SFPs [23]. This is especially true after mating when it is necessary to quickly replace SFPs that had been transferred to the female [24]. Consistent with its role as a major secretory tissue, the AG shows one of the highest basal levels of ER stress, as indicated by fluorescent reporters of upstream ER stress signals[9,10]. We show here, by gene expression analysis, that genes commonly upregulated in response to ER stress in *Drosophila* [18] are also most highly expressed in the AG.

We further hypothesized that induction of excessive levels of ER stress, above the already high basal levels, would, however, perturb AG function. To induce excessive levels of ER stress in the AG, we conditionally expressed a misfolded protein in this tissue. To induce high levels of ER stress by an independent genetic mechanism, we knocked down *BiP* expression in the AG. Both perturbations induced further UPR signaling in the AG, as indicated by increased *Xbp1* splicing. Both genetic manipulations also resulted in male infertility. The AGs in both models were smaller than controls and their lumens appeared empty. Correspondingly, their SFP protein levels were dramatically decreased. Sperm production, motility, and transfer appeared to be unaffected by excessive ER stress in the AG. In addition to providing information about *Drosophila* AG biology, the methods put forth in this study provide novel tools for studying insect secretory tissues such as reproductive glands, including in insects where genetic tools are more limited.

## Results

### ER stress-responsive genes are upregulated in the normal AG

**Fig 1:**
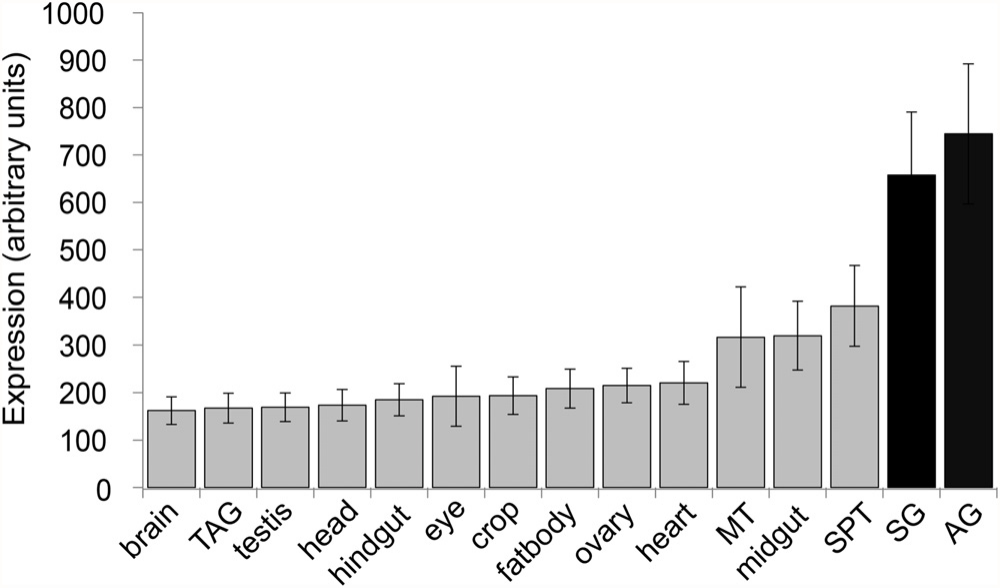
Expression profile of ER stress-responsive genes. ER stress responsive genes (ERSRGs) [18] are most highly expressed in the accessory gland and salivary gland (p<0.01). Displayed are average expression values of 487 ERSRGs for adult tissues. All expression data are taken from Flyatlas.org [27]. Expression is measured in arbitrary units, normalized across tissues [27]. Mean +/- SE. TAG: thoracicoabdominal ganglion; SPT: spermathecae; MT: malpighian tubules; AG: salivary gland; AG: accessory gland. SG and AG are highlighted black for emphasis.

*Xbp1* mRNA splicing is a classic sign of the presence of ER stress and the induction of the UPR [25,26]. Under ER stress conditions, *Xbp1* mRNA undergoes unique cytoplasmic splicing by IRE1 [25,26]. The *Xbp1* spliced product is translated and acts as a transcription factor to turn on UPR genes. An increase in the amount of *Xbp1* spliced product is a clear sign of UPR induction [25,26]. In healthy unstressed *Drosophila* m*a*les, *Xbp1* mRNA splicing and expression has been shown to be highest in the male AG, compared to all adult tissues [9,10], suggesting that the AG normally experiences high levels of ER stress. To further test whether the AG is undergoing high basal levels of ER stress, we examined the expression of genes whose expression levels are induced by ER stress. We previously identified 487 ER stress-responsive genes (ERSRG) by profiling genetic variation in *Drosophila* [18]. These genes are enriched for GO categories related to ER stress response and the UPR. We compared the expression patterns of these ERSRG, as measured by FlyAtlas [27]. We find that, as a group, the ERSRG are most highly expressed in secretory tissues such as the salivary gland (SG) and the AG (t-test, p<0.01; Fig. 1). Other secretory tissues, including the female spermatheca, the midgut, and the malpighian tubules express moderate levels of ERSRG, but levels are at least two-fold lower than in the AG.

### Genetic induction of the UPR in the AG

Reasoning that the AG might be tolerant of high levels of ER stress, we took two different genetic approaches to induce excessive levels of ER stress and the UPR in the AG. To achieve AG-specific expression of the UPR inducers, we drove expression with the *prd-GAL4* driver [28]. This well characterized driver is expressed in the primary and secondary cells of the AG and has been used previously to drive high levels of gene expression, and of RNAi for knockdown, specifically in the male AG [22,29,30,31]. *prd-GAL4* expression occurs during the development of the AG, in the adult AG, and is induced by mating [28]. *prd-GAL4* is not expressed in the testes and does not affect testis development [28].

**Fig 2:**
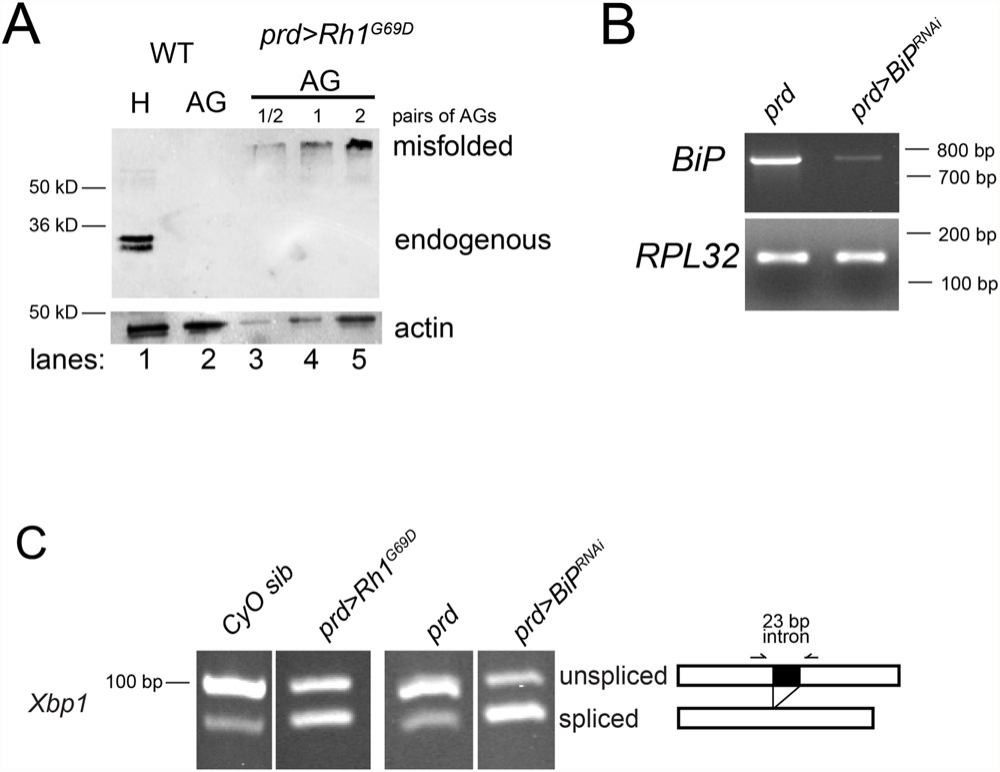
Genetic induction of ER stress and UPR in the AG. A) Western blot analysis of endogenous and misfolded Rh1 expression. Endogenous Rh1 is normally expressed only in the eyes. A sample of head tissue (with eyes) is included as a reference sample to validate the specificity of the antibody. Expression of endogenous Rh1 is present in the head sample (lane 1: H = protein from one head) and not the wild type AG sample (lane 2: AG = protein from two pairs of AG). Misfolded Rh1 is expressed in AG from *prd>Rh1* ^*G69D*^ flies (lanes 3–5). The high molecular weight Rh1 band is likely due to aggregation of the misfolded protein. Actin was a loading control. *prd>Rh1* ^*G69D*^ AG lanes 3–5: half, one, and two pair(s) of AG. Bands of expected sizes were observed; size measurements are based on the Invitrogen SeeBlue Plus2 Protein Standard. B) RNAi knockdown of *BiP* mRNA in AG is nearly complete. RT-PCR of *BiP*, and *Rpl32* control, transcripts from AG cDNA of control *prd* and *prd>BiP* ^*RNAi*^ male flies. Bands of expected sizes were observed based on an Invitrogen 100 bp DNA ladder. C) Splicing of *Xbp1* is increased in AG from *prd>Rh1* ^*G69D*^ and *prd>BiP* ^*RNAi*^ flies as compared to AG from CyO sibling and *prd* control flies, respectively. The diagram on the right indicates position of primers used for amplification and the 23 bp difference due to the unconventional splicing of *Xbp1* transcript by IRE1 (unspliced = 100 bp and spliced = 77 bp). Bands of expected sizes were observed; size measurements are based on an Invitrogen 100 bp DNA ladder. All analysis was performed on 3–5 day old virgin males. Each panel of the figure is representative of independent experiments performed in triplicate.

First, we ectopically expressed a mutant form of the *Drosophila* Rh1 protein. The *Rh1* ^*G69D*^ mutation results in a misfolded rhodopsin protein that induces the UPR when expressed in the retina [8]. We hypothesized that expression of the Rh1^G69D^ misfolded protein in the AG would also strongly induce the UPR in the AG by flooding and overwhelming the ER with ectopic misfolded protein [8]. We use *prd-GAL4* to express *Rh1* ^*G69D*^ (*UAS-Rh1* ^*G69D*^) in an AG specific manner (*prd>Rh1* ^*G69D*^). This results in strong expression of the misfolded *Rh1* ^*G69D*^ protein in the AG (Fig. 2A).

Second, to constitutively induce the UPR in a way that did not involve the product of an exogenous misfolded protein, we knocked down *BiP* (*Hsc70–3*) expression in the AG. We used the *prd-GAL4* driver to express an RNAi construct specific to *BiP* (*prd>BiP* ^*RNAi*^). This resulted in knockdown of *BiP* transcript in the AG (Fig. 2B).

To determine if the UPR is activated in the AG of *prd>Rh1* ^*G69D*^ and *prd>BiP* ^*RNAi*^ flies, we measured the splicing of *Xbp1* mRNA. We find that *Xbp1* mRNA splicing is strongly induced in the AG of *prd>Rh1* ^*G69D*^ and *prd>BiP* ^*RNAi*^ flies as compared to control *prd* flies (Fig. 2C), indicating the further induction of ER stress and the UPR above basal levels.

### Induction of UPR ablates AG function

**Fig 3:**
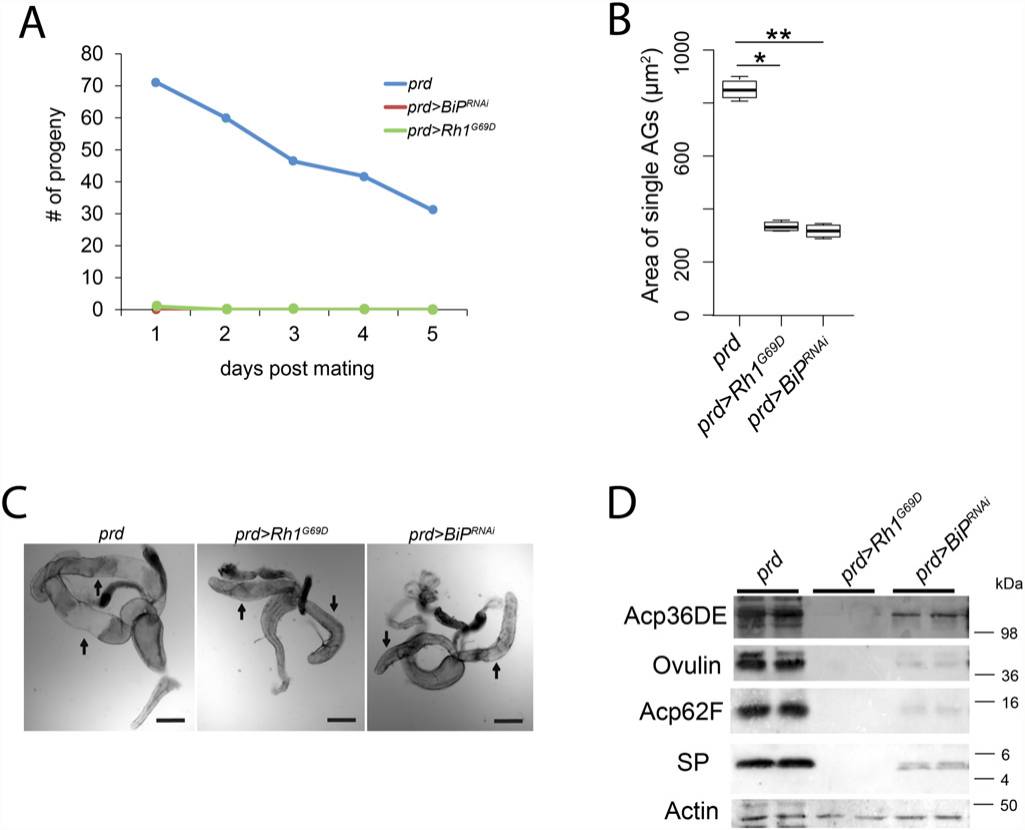
Induction of UPR ablates AG functions. A) *prd>Rh1* ^*G69D*^ and *prd>BiP* ^*RNAi*^ male flies are infertile (N = 20 for all genotypes). Control *prd* male flies show normal fertility. Males of all three genotypes were mated to Canton S females. B) AGs from *prd>Rh1* ^*G69D*^ (** p = 4.6x10^-16^) and *prd>BiP* ^*RNAi*^ (* p = 7.5x10^-16^) males are smaller than AGs from control *prd* males, similar to previous studies that ablate SFP production in the AG [32]. Mean ± SD. N = 8 for all genotypes. C) Male reproductive tracts from *prd* control, *prd>Rh1* ^*G69D*^, and *prd>BiP* ^*RNAi*^ male flies. AGs from *prd>Rh1* ^*G69D*^, and *prd>BiP* ^*RNAi*^ male flies are narrow and their lumens appear to be empty as compared to the AG from *prd* control male flies. Arrows point to AG. Testes were removed from these male reproductive tracts for clarity. Other reproductive tract tissues (ejaculatory duct and seminal vesicles) appear normal in the *prd* control, *prd>Rh1* ^*G69D*^, and *prd>BiP* ^*RNAi*^ males and are not labeled in the figure. Scale bar: 200 μm. D) Complete and nearly complete loss of SFP protein in AG from *prd>Rh1* ^*G69D*^ and *prd>BiP* ^*RNAi*^ males, respectively, relative to controls (*prd*). Each lane represents one pair of AG. Actin is the loading control. Bands of expected sizes were observed based on the Invitrogen SeeBlue Plus2 Protein Standard. Detection of SFPs and actin are from a single blot. All data shown are representative of experiments performed in independent triplicate experiments. All analyses were performed on 3–4 day old virgin males.

To assess the effect of ER stress and UPR induction on the AG, we undertook several measures of AG function. Because the AG produces SFPs and the production of SFPs is critical to male fertility [28,32] we tested the fertility of *prd>Rh1* ^*G69D*^ and *prd>BiP* ^*RNAi*^ male flies. We found that *prd>Rh1* ^*G69D*^ (N = 20) and *prd>BiP* ^*RNAi*^ (N = 20) males were infertile (Fig. 3A and S1 Table). Females mated to either genotype produced no progeny over five days post mating. Control *prd* males (N = 20) had the expected level of fertility, producing more than 200 progeny over five days of egg laying.

The AG of *prd>Rh1* ^*G69D*^ or *prd>BiP* ^*RNAi*^ male flies were approximately two and half times smaller in area and narrower than those of control *prd* males (p<4.6x10^-16^; Fig. 3B and C, S2 Table), consistent with their lumens being greatly depleted of secretions. Western blot analysis demonstrates that the four SFPs examined (Acp36DE, Ovulin, Acp62F, and SP) were undetectable in AGs from *prd>Rh1* ^*G69D*^ and nearly undetectable in AGs from *prd>BiP* ^*RNAi*^ males (Fig. 3D).

**Fig 4:**
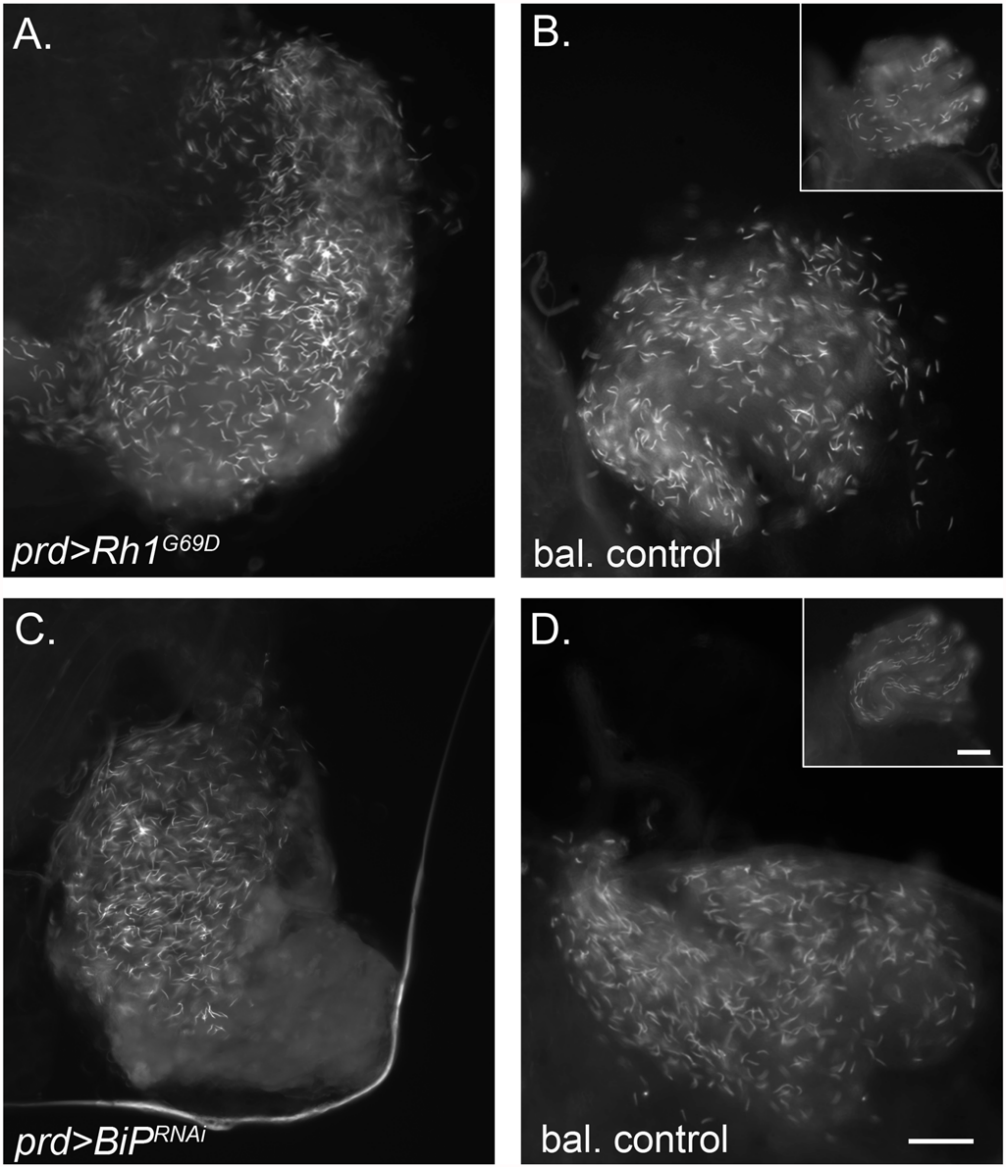
Sperm transfer is normal in flies with UPR induced in the AG. *prd>Rh1* ^*G69D*^ and *prd>BiP* ^*RNAi*^ males transfer wild-type levels of sperm. Female reproductive tracts containing transferred, GFP-labeled sperm from *prd>Rh1* ^*G69D*^ (A) and *prd>BiP* ^*RNAi*^ males (C) and their respective balancer sibling controls (bal. control; B, D) (scale bar = 50 μm). Insets in B and D show sperm stored in the seminal receptacle which does not occur in either *prd>Rh1* ^*G69D*^ and *prd>BiP* ^*RNAi*^ males likely due to loss of sperm-storage promoting AG proteins like Acp36DE [46] (scale bar = 100 μm). We examined multiple tracts from each cross (*prd>Rh1* ^*G69D*^: N = 10, bal. control: N = 10; *prd>BiP* ^*RNAi*^: N = 9, bal.control: N = 8) and present representative photographs of sperm transferred by normal males or males whose AG had been ER-stressed. We are unable to obtain precise sperm-counts of sperm because of the very high numbers transferred by male Drosophila (~2000–4000; [41,44,45]) coupled with the fact that it is impossible to spread the sperm out evenly to count them. The latter is even more severe in the females who did not receive SFPs, since these females’ uteri fail to expand due to the lack of SFPs [47], resulting in the sperm mass becoming mixed with male secretions that will form the mating plug (Mattei et al., in preparation).

We eliminated male fertility by perturbing AG function and this result agrees with previous studies that eliminate AG function and observed male fertility [32,33]. To confirm that male infertility of these flies was due to abnormal AG function, we examined sperm transfer by *prd>Rh1* ^*G69D*^ and *prd>BiP* ^*RNAi*^ males, relative to control *prd* males. We find that *prd>Rh1* ^*G69D*^ and *prd>BiP* ^*RNAi*^ males transfer normal amounts of sperm to females (Fig. 4), indicating that sperm production, or transfer are not affected by expression of Rh1^G69D^ or knockdown of BiP in the AG. The sperm were also normally motile (data not shown). Thus, Rh1^G69D^ or *BiP* RNAi driven by *prd-GAL4* does not affect the testes, consistent with previous reports that *prd-GAL4* is not expressed in the testes [28]. Together these data demonstrate that strong induction of UPR ablates the function of the AG and results in male infertility.

## Discussion

We report that AG-specific expression of a misfolded protein or AG-specific knockdown of *BiP* expression in *Drosophila melanogaster*, results in impaired AG function and male infertility. While the AG shows high basal levels of ER stress and UPR induction, both genetic manipulations that we performed are predicted to further increase ER stress in the AG. Indeed, we found that both genetic manipulations increased *Xbp1* mRNA splicing above basal levels in the AG, consistent with increase ER stress levels. Our study demonstrates that induction of excessive ER stress and the UPR interferes with AG function. The AG might naturally experience excessive ER stress for various reasons. For example, mutations that result in a mutant misfolded SFP or result in a mutant SFP that is retained in the ER could impair male fertility by further inducing the UPR in the male AG.

We observed AG-specific expression of a misfolded protein or knockdown of *BiP* expression resulted in near elimination of SFP expression. There are several possible scenarios for why this is observed. First, loss of SFP protein expression could result from the attenuation of translation that occurs because of signaling through the PERK UPR pathway [34]. Second, impairment of SFP expression could be due to the phenomenon known as regulated Ire1-dependent decay (RIDD) [35]. RIDD occurs when the UPR is induced and IRE1 actively degrades transcripts that are dependent on the ER for translation and processing (i.e. all SFPs). Future studies are needed to determine if these or other mechanisms are responsible for the loss of SFP expression in ER stressed AG.

The approaches in this study can be extended to other tissues in *Drosophila*. Induction of ER stress by targeted expression of a misfolded protein complements the common pharmacological methods to induce ER stress [8,18,36], and offers some advantages. Genetic expression of a misfolded protein allows for studies targeted to both tissue and developmental time points. The ease of expressing the *Rh1* ^*G69D*^ allele in a tissue specific manner makes it an ideal tool for inducing ER stress. For example, we have also expressed the *Rh1* ^*G69D*^ allele in the male ejaculatory bulb (EB), another reproductive organ with a heavy secretory load, and found loss of EB function due to elimination of the normal secreted protein products of this tissue (Avila et. al, in prep).

AG-specific expression of *Rh1* ^*G69D*^ represents a novel tool for studying AG and SFP function. Our result agrees with previous studies that eliminated AG function through the use of targeted delivery of diptheria toxin A [32] or through the use of a *prd* mutant/transgene combination [33]; both studies also observed male infertility. Each of these previous methods to genetically eliminate AG function has drawbacks compared to the method we used, particularly when considering its potential for use in less genetically-tractable organisms. For example, in the study utilizing diphtheria toxin A [32], leakage of the *D. melanogaster* driving promoter resulted in low levels of toxin production in the testes, thus eliminating sperm production, in some cases. The *prd* mutant and transgene combination used to eliminate AG function in the other study would be difficult to replicate in other organisms [33]. The method reported here, of using directed expression of a misfolded protein to eliminate AG function, avoids these drawbacks. Because the UPR is so well conserved, our approach can be extended to impair accessory gland function in other insects, if an AG-specific promoter (e.g. in *Aedes aegypti* [37]) is available to drive expression of the misfolded protein. Thus, this may provide a general method applicable to the control of fertility of insect vectors of human disease.

## Materials and Methods

### Expression analysis

The 487 ER stress responsive genes (ERSRG) were from Chow et al., 2013 [18]. Expression levels were taken from FlyAtlas [27] for analysis. Only adult tissues were considered. T-tests were used to test for enrichment in secretory tissues as compared to other adult tissues. Statistical analysis was performed in R (www.R-project.org/).

### *Drosophila* cultures

Flies were reared under standard laboratory conditions on agar–dextrose–yeast medium at 24°C on a 12-h light/dark cycle. All experimental male flies were 3–4 day old virgins. The *paired-GAL4* (*prd-GAL4*) driver line was from the Bloomington Stock Center (stock 1947) [28]. The *BiP* RNAi line and attp (II) lines were purchased from the Vienna *Drosophila* RNAi Center (BiP: transformant ID: 14882; attp (II): transformant ID 60100). Control *prd* flies are non-balanced progeny from a cross between *prd-GAL4* driver and the *attp* (II) line. *UAS-Rh1* ^*G69D*^ flies were a generous gift from Dr. H.D. Ryoo [8]. Standard 3–4 day old Canton S females were used for fertility tests.

### RT-PCR

Total RNA was extracted from 3–4 day old virgin male AG using the TRIzol reagent (Invitrogen) and stored at -80°C (as in [38]). cDNA was synthesized from equivalent amounts of cDNA for each sample using an Invitrogen kit. *BiP*, *Rpl32*, and *Xbp1* were all amplified by standard RT-PCR protocols. *BiP* and *RPL32* PCR products were visualized on a 2% agarose gel. *Xbp1* PCR products were visualized on a 12% acrylamide gel to resolve the 23 bp difference between splice products. Using the following primers the *Xbp1* unspliced product is 100 bp and the spliced product is 77 bp: Forward—TCAGCCAATCCAACGCCAG and Reverse—TGTTGTATACCCTGCGGCAG. The size markers in Fig. 2B and Fig. 2C are from a 100 bp ladder (Invitrogen). All expression analyses were performed in triplicate independent experiments. Representative images are shown in the figures.

### Western blots

Proteins were extracted from 3–4 day old virgin male AG or heads (as in [38]). Western blotting for Acp36DE, Ovulin, Acp62F, sex peptide (SP), and Actin was performed as previously described [39]. Western blotting for Rh1 was as previously described [40]. The Rh1 monoclonal antibody (4C5) was purchased from the Developmental Studies Hybridoma Bank (University of Iowa). The size markers in Fig. 2A and Fig. 3D are from the SeeBlue Plus2 Protein Standard (Invitrogen). All Western blot analyses were performed in triplicate independent experiment. Representative images are shown in the figures.

### Fertility tests

Fertility tests were performed with 3–4 day old virgin males. Males of all three genotypes were mated to Canton S females in single pairs. Matings were observed, and males were discarded after the single mating. Females were allowed to lay eggs in a vial for 24 hrs. and then transferred to a new vial each day for five days. Females were discarded after the fifth day post-mating. Progeny that emerged were counted.

### AG measurement

AG measurements were performed on 3–4 day old virgin males. Male reproductive tracts were dissected and visualized under phase contrast using a Leica DM 500B fluorescence microscope (Leica Microsystems). The area of each lobe of the AGs was measured using ImageJ software (www.http://imagej.nih.gov/ij/). Four pairs of AGs were measured for each genotype (eight total lobes). Statistical analysis was performed with t-tests in R (www.R-project.org/). Representative images are shown in fig. 3C.

### Sperm transfer

To visualize transferred sperm in *prd>Rh1* ^*G69D*^ and *prd>BiP* ^*RNAi*^ males, we generated a *prd*-GAL4 line that contains a protamine-GFP transgene [41]. *protamineB*-GFP; +/+; *prd*-GAL4/TM3, *Sb*, *Ser* females were crossed to UAS-*Rh1* ^*G69D*^/CyO or UAS-*BiP* ^*RNAi*^ males to generate *prd>Rh1* ^*G69D*^ and *prd>BiP* ^*RNAi*^ males with GFP-labeled sperm nuclei. Balancer siblings from each cross were used as controls. Experimental and control males were mated to wild-type Canton S females. Males and females were collected within 4 hours of eclosion and aged for 3–5 days in vials containing yeast-glucose medium prior to examination. Females mated to experimental and control males were flash frozen in liquid nitrogen at 35 min post-mating, and their uteri dissected in 1X PBS and visualized using a Leica DM 500B fluorescence microscope (Leica Microsystems). We examined multiple tracts from each cross (*prd>Rh1* ^*G69D*^: N = 10, bal. control: N = 10; *prd>BiP* ^*RNAi*^: N = 9, bal.control: N = 8). Due to large numbers of sperm transferred (~2000–4000) and size and shape of the uteri, it is not possible to count sperm [42,43,44,45]. However, we see examined sperm motility in *prd>Rh1* ^*G69D*^ males: we dissected 3–5 day old male reproductive tracts and ruptured their seminal vesicles on a slide with a coverslip. We detected no differences in sperm motility (not shown) between *prd>Rh1* ^*G69D*^ (N = 5) and control males (N = 7).

## Supporting Information

S1 Table Progeny counts. (XLSX)

S2 Table Accessory gland size measurements. (XLSX)
